# Research progress of exosomes in pathogenesis, diagnosis, and treatment of ocular diseases

**DOI:** 10.3389/fbioe.2023.1100310

**Published:** 2023-01-24

**Authors:** Xinting Feng, Zhen Peng, Lingyi Yuan, Ming Jin, Haijian Hu, Xin Peng, Yaohua Wang, Chun Zhang, Zhiwen Luo, Hongfei Liao

**Affiliations:** ^1^ Jiangxi Provincial Key Laboratory for Ophthalmology, Jiangxi Clinical Research Center for Ophthalmic Disease, Affiliated Eye Hospital of Nanchang University, Jiangxi Research Institute of Ophthalmology and Visual Science, Nanchang, China; ^2^ Department of Sports Medicine, Huashan Hospital, Fudan University, Shanghai, China; ^3^ Department of Sports Medicine, Shanghai General Hospital, Shanghai Jiao Tong University, Shanghai, China; ^4^ College of Fine Arts, Jiangxi Science and Technology Normal University, Nanchang, China; ^5^ Department of ophthalmology, West China hospital, Sichuan University, Chengdu, China

**Keywords:** exosome, extracellular vesicle, stem cell, ocular disease, engineered exosome, treatment

## Abstract

Exosomes are natural extracellular vesicles with a diameter of 30–150 nm, which exist in biological fluids and contain biomolecules related to the parent cell, such as proteins, nucleic acids, lipids, etc. It has a wide range of biological functions, and participates in the regulation of important physiological and pathological activities of the body. It can be used as a biomarker for early diagnosis of ocular diseases, a potential therapeutic target, a targeted drug carrier, and has a high potential for clinical application. In this paper, we summarized the genesis mechanism, biological functions, research and application progress of exosomes, focused on the engineering strategy of exosomes, and summarized the advantages and disadvantages of common engineering exosome preparation methods. Systematically combed the role of exosomes in corneal diseases, glaucoma, and retinal diseases, to provide a reference for further understanding of the role of exosomes in the pathogenesis, diagnosis, and treatment of ocular diseases. Finally, we further summarized the opportunities and challenges of exosomes for precision medicine. The extension of exosome research to the field of ophthalmology will help advance current diagnostic and therapeutic methods. Tiny exosomes have huge potential.

## Introduction

Exosomes are lipid bilayer extracellular vesicles (EVs) secreted by various cells, with a diameter of 30–150 nm. They are the smallest subset of extracellular vesicles, distributed in various biological fluids, tears, aqueous humor, vitreous and blood are rich in exosomes (Liu et al., 2020). The exosomes were first discovered in sheep reticulocytes by Pan and Johnstone (1983). They were originally used to describe the membrane vesicles released by reticulocytes during differentiation, during which transferrin receptors are lost and exosomes are produced. Exosomes contain a variety of contents, the composition of which is different according to the type of cells from which they originate and the extracellular environment, resulting in a wide range of biological functions, including cellular communication, cellular waste management, inflammation regulation, immune regulation, and repair and regeneration. Meanwhile, the lipid composition of exosomes is of great significance for their stability in the extracellular space, protecting their contents from degradative enzymes or other chemicals in the extracellular environment, and has excellent stable chemical properties (Skotland et al., 2019).

Ocular diseases bring great inconvenience to people’s daily life, and some of them have insidious early symptoms. The complex anatomical structure of the eye and the existence of physiological barriers bring great challenges to the early diagnosis and treatment of eye diseases (Weng et al., 2017; Liang et al., 2018). Exosomes have been shown to be involved in the pathogenesis of ocular diseases, and sometimes the changes of exosome cargo can reflect the changes of ocular diseases, so as to be used as diagnostic markers. As a natural carrier, exosomes have higher barrier-crossing ability and safety than synthetic nano-drug carriers, and are more capable of delivering diverse drugs or bioactive molecules. In this article, we take representative ophthalmic diseases with different levels of anatomy (keratonosus, glaucoma, retinal diseases) to elucidate the roles of exosomes from different sources in their pathogenesis, diagnosis, and treatment. Exosomes can provide therapeutic effects to ocular diseases by signaling or transporting biomolecules, which can serve as ideal drug delivery vehicles for targeted therapies. We also summarized important information about exosomes, including their biogenesis, composition, biological functions, isolation and purification methods, and engineering strategies.

## Systematic description of exosomes

### Biogenesis and uptake of exosomes

EVs can be classified into three types, according to their biological origin and size: exosomes (30–150 nm in diameter), microvesicles (100–1,000 nm), and apoptotic bodies (>1,000 nm) (Manukonda et al., 2022). Their diameters differ greatly, and their biogenesis is different, with different characteristics. Exosomes are derived from endosomes, the cell membrane invaginates to form early endosomes, the early endosomes mature into late endosomes and then transform into multivesicular bodies, which contain intraluminal vesicles (future exosomes). Eventually, the multivesicular bodies fuse with the cell membrane and release the exosomes into the extracellular space through exocytosis (Kalluri and LeBleu, 2020). The molecular mechanism of exosome genesis has not been fully elucidated, but studies have shown that the endosomal sorting complex required for transports (ESCRT) (Colombo et al., 2013), tetraspanins (van Niel et al., 2011), ceramides (Trajkovic et al., 2008), and lipids (Skotland et al., 2019) are all involved in exosome genesis, to achieve specific sorting of exosome contents (Zhang Y. et al., 2019).

**FIGURE 1:**
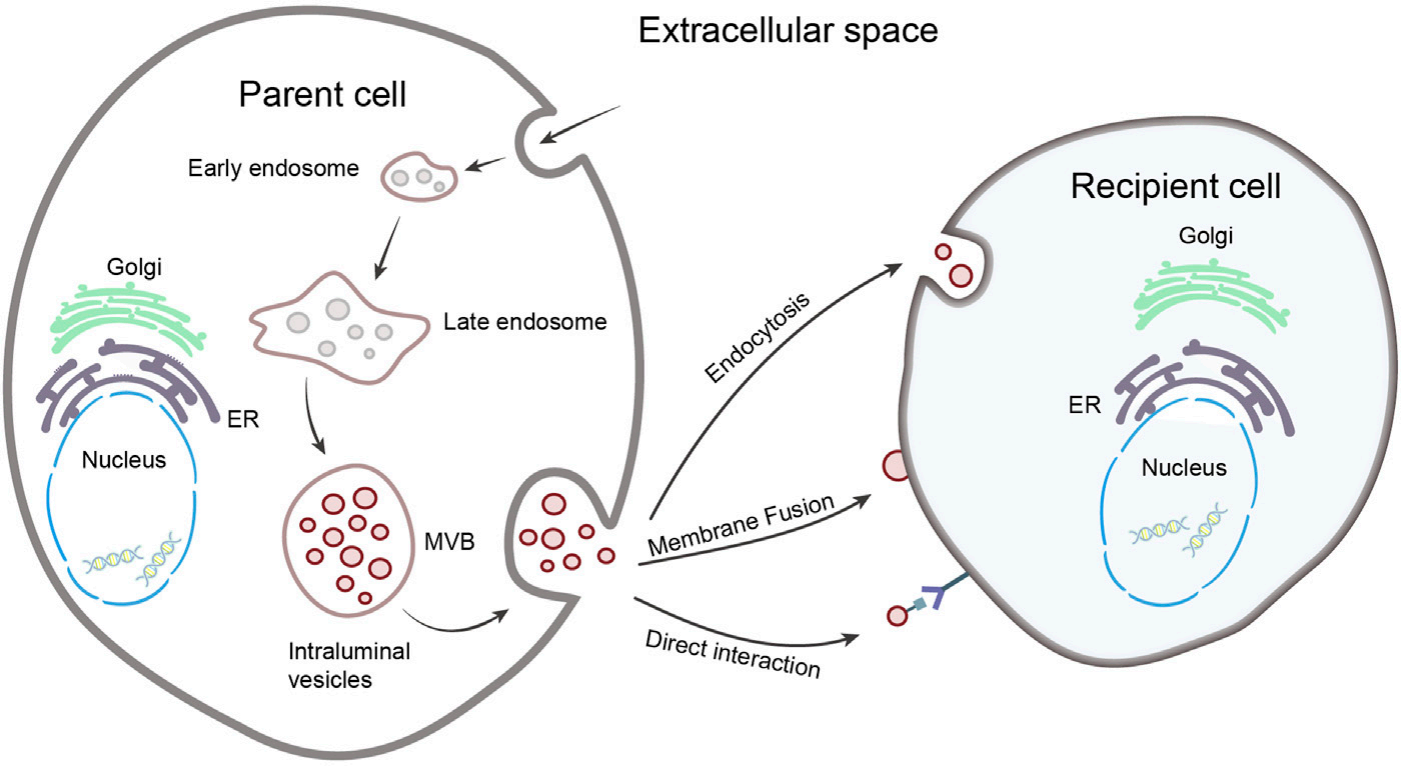
Biogenesis and uptake of exosomes. The parent cell membrane invaginates to form early endosome, matures to late endosome, and transforms into multivesicular body (MVB), contain intraluminal vesicles. The MVB fuse with the cell membrane and release exosomes. After exosomes are released, information can be transmitted to recipient cells through membrane fusion, direct interaction and endocytosis.

After being secreted by the cell, exosomes can integrate their contents into the recipient cell by fusing with the cell membrane; or the various forms of endocytosis, such as endocytosis mediated by lipid raft, clathrin, caveolin, and phagocytosis, macropinocytosis, deliver the various messages to the recipient cells; it can also communicate with target cells directly through receptor-ligand interaction (Figure 1) (Gurung et al., 2021). It has not been determined whether different exosome uptake patterns in recipient cells result in different functional effects.

### Composition and biological functions of exosomes

**FIGURE 2:**
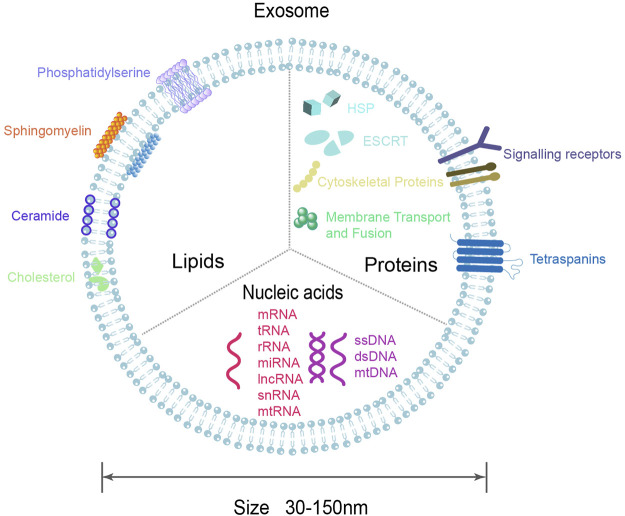
Diagram of exosomal molecular composition. Exosomes are composed of lipid bilayers that carry a variety of biomolecules, including proteins, DNA, RNA, lipids, etc.

Exosomes of different cellular origins differ in size, contents, and effects on recipient cell function, showing high heterogeneity (Chiang and Chen, 2019; Kalluri and LeBleu, 2020). Exosomes contain many components of parent cells, includes growth factors, cytokines, signaling lipids, mRNA, miRNA and proteins (Phinney and Pittenger, 2017). So far, 9,769 proteins, 3,408 mRNAs, and 2,838 miRNAs have been identified in exosomes (exocarta.org). Exosomes are rich in proteins, including non-specific and specific proteins (Doyle and Wang, 2019; Mashouri et al., 2019; Gurung et al., 2021). The non-specific proteins are independent of the cellular origin of the exosomes, which are present in all exosomes, such as ESCRT proteins (Alix, TSG101), heat shock proteins (HSC70, HSC90), transport and fusion proteins (Rab protein, Annexins, Flotillin), cytoskeletal proteins (Actin, Tublin, Cofilin), can be used to detect the presence of exosomes. In contrast, specific proteins are associated with exosome-derived cells, such as MHC Class I and MHC Class II proteins, transferrin receptors, which can reflect the specificity of tissue and cell, and may mediate signaling. Exosomes contain a variety of lipids, including sphingolipid, cholesterol, ceramide, etc., which not only maintain chemical stability, but also participate in exosome biogenesis (Minciacchi et al., 2015). In addition, the exocrine cavity also contains a variety of nucleic acids, including DNA (single-stranded DNA, double-stranded DNA) and RNA (miRNA, lncRNA, mRNA, rRNA, etc.), among which miRNA is the most abundant (Figure 2) (Gurung et al., 2021). The composition of exosomes may reflect not only the composition of the parent cells to some extent, but also the existence of different sorting mechanisms during exosome biogenesis.

The biological functions of exosomes are extensive, and the understanding of their biological functions helps to study the role of exosomes in health and disease. 1) Signal transduction: the most basic and important biological function of exosomes. As carriers of cellular communication, exosomes can transport their contents to nearby or distant cells, thus regulating various physiological and pathological processes (Wei et al., 2021). 2) Inflammatory regulation: exosomes can promote/inhibit the activation of inflammasomes (Noonin and Thongboonkerd, 2021). Luo Z. W. et al. (2021); Luo et al. (2022a) demonstrated that exosomes secreted by cells in an inflammatory environment can induce M1 polarization and inhibit M2 polarization of macrophages, which in turn promote the inflammatory response. 3) Immunomodulation: immune cell-derived exosomes can promote/inhibit the immune response, depending on the composition of exosomes and the induced cellular environment, allowing exosomes to play an important role in immune response and immune privilege (Li Q. et al., 2019). 4) Repair and regeneration: mesenchymal stem cell (MSC) derived exosomes play a protective and therapeutic role in wound healing and tissue regeneration (Bakhtyar et al., 2017; Chen W. et al., 2021). 5) Autophagy regulation: exosomes from different sources can promote/inhibit autophagy (Hassanpour et al., 2020). In addition, exosomes can be used as biomarkers and therapeutic carriers of diseases. The composition of functional molecules in exosomes varies according to their secreting cells and is influenced by the cellular microenvironment. Different functional molecular compositions allow for a wide range of exosome biological functions (Gurunathan et al., 2019).

### Isolation and purification of exosomes

Exosomes are widely found in various biological fluids as well as cell culture supernatants (Yanez-Mo et al., 2015). Common techniques for exosome isolation include ultracentrifugation, density gradient centrifugation, ultrafiltration, size exclusion chromatography, chemical precipitation, immunoaffinity capture, and microfluidic techniques (Chiang and Chen, 2019; Sun et al., 2022). Ultracentrifugation is the most common technique and the gold standard for exosome separation (Li et al., 2017). Density gradient centrifugation can be considered as an advanced version of ultracentrifugation, which can obtain exosomes with high purity. However, this method is cumbersome and has a relatively low yield (Van Deun et al., 2014). Ultrafiltration, one of the simplest separation techniques, passes exosomes through filtration membranes with different molecular weight cutoff. Size exclusion chromatography is similar to ultrafiltration (Chen J. et al., 2021). The principle of chemical precipitation is protein coagulation, which is easy to operate. However, the exosomes obtained are of low purity and often contain macromolecular protein impurities which are not conducive to proteomic analysis of exosomes. The principle of immunoaffinity capture is an antigen-antibody-specific reaction. The exosomes obtained by this method have high purity but low activity (Greening et al., 2015). Microfluidic chip separates exosomes according to physical and biochemical characteristics (Chen W. et al., 2021). This method is characterized by high speed, high throughput, and strong specificity. It can rapidly detect exosomes from a large number of clinical samples, which is a new diagnostic technology with promising clinical applications.

Different methods of isolation and purification have their advantages and disadvantages, but there is no technology available to isolate exosomes to absolute purity (Thery et al., 2018). In addition to developing new technologies, the combined application of the above techniques may help to overcome the shortcomings of individual technology, and give consideration to the yield and purity as much as possible without damaging the bioactivity of exosomes. With the continuous development of exosome isolation and purification technology, the application of nanotechnology and microfluidic chips can optimize the study of exosomes. Standardization and new exosome isolation protocols can help improve the consistency of exosome studies, and the application of exosomes in the field of ophthalmology will continue to expand.

### Strategies for engineering exosomes

Natural exosomes still face some challenges as therapeutic molecular delivery systems (Jafari et al., 2020). The therapeutic effect of natural exosomes is limited due to the absence of pharmaceutical components. Animal experiments have demonstrated that natural exosomes have poor tissue and cell targeting ability (Tian et al., 2018), and their cargo will be non-selectively transported to the recipient cells. Fortunately, exosomes are highly engineerable, namely engineered exosomes, to further improve their targeting, safety, therapeutic efficiency, and loading of more effective functional molecules to suit clinical needs (Zhang et al., 2021).

**FIGURE 3:**
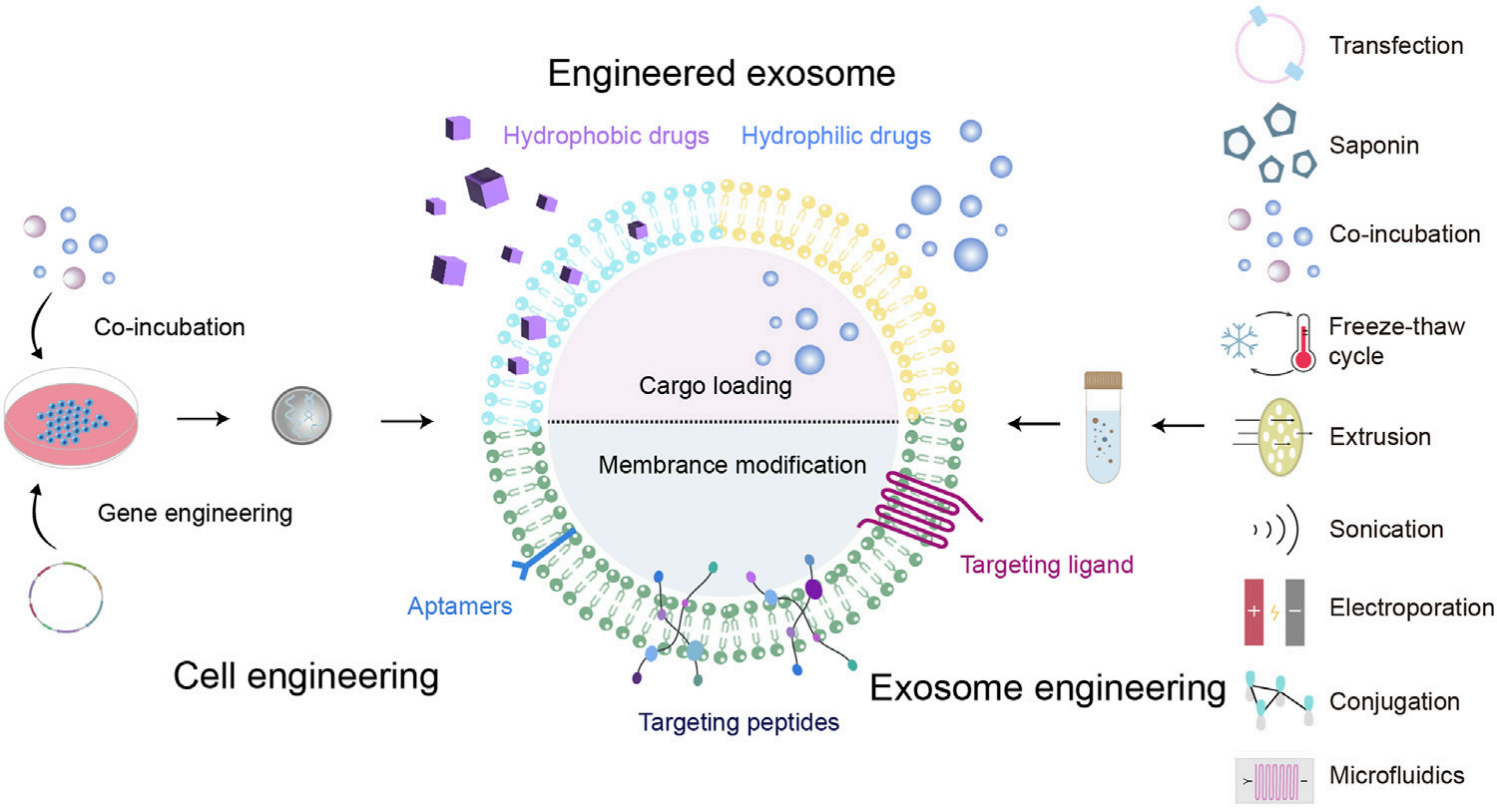
Diagram of engineered exosome. The engineering strategies of exosomes include cell engineering and exosome engineering. Hydrophobic drugs, hydrophilic drugs and targeted ligands can be loaded onto exosomes through different methods.

Currently, exosome engineering strategies are divided into “cell engineering” and “exosome engineering” in terms of time (Figure 3) (Lu et al., 2018). “Cell engineering” is to use genetic engineering and co-incubation to transform parent cells, transfect nucleotides or import proteins, and load them into exosomes. While “exosome engineering” is the modification of exosomes directly after they are secreted. It also can be divided into exosome membrane modification and content loading in space (Xu et al., 2020). The main purpose of outer membrane modification is to enhance the targeting of exosomes, reduce the clearance of exosomes, increase the concentration of exosomes at the disease site, maximize the therapeutic effect and reduce cytotoxicity and adverse reactions (Liang et al., 2021). While content loading can make exosomes load specific nucleic acids, proteins, small molecule drugs, etc. Zhang et al. (2022) used extrusion and incubation methods to treat neutrophil exosomes, and constructed engineered exosomes modified with superparamagnetic iron oxide nanoparticles and loaded with chemotherapeutic drugs. Under the action of the external magnetic field, the exosomes were targeted and enriched at tumor sites, achieving a dual effect of tumor treatment, almost completely eliminating tumor growth, and greatly prolonging the survival time of tumor mice. In addition, biocompatible scaffolds loaded with exosomes can be developed to construct exosome carriers, which can deliver exosomes in targeted batches. Achieve sustained and controlled release of exosomes, and enhance its therapeutic effect (Mishra et al., 2021). In another study, three different loading procedures, incubation, sonication, and electroporation, were used and the results proved that sonication was the most efficient (Kim et al., 2016). The engineering strategy of exosomes and the construction of exosome carriers, combining low immunogenicity, nanoparticles, targeted drug delivery, and drug delivery systems, are helpful to improve the half-life of exosomes and enable targeted enrichment, which is important for biomedical research and clinical translation of exosomes. Further, Table 1 summarizes the advantages and disadvantages of common engineering strategies.

**TABLE 1:** Advantages and disadvantages of exosome engineering strategies.

Engineered exosome strategy	Operation method	Advantages	Disadvantages	References
Gene engineering	Genetic modification of secretory cells using viral or non-viral vectors	Maintains the integrity of the membrane structure of exosome	Cumbersome operation and high technical requirements	Mishra et al. (2021)
Chemical transfection	Using transfection reagents (such as liposome 2000) to form holes in the exocrine membrane	Effective and load efficient	May damage the structural integrity and stability of exosomes; incomplete removal of transfection reagents	Stickney et al. (2016)
Saponin	Saponins are surfactants that cause exosome membranes to form small holes	Maintain the integrity of the exocrine structure	Residual saponins need to be removed in a subsequent purification step	Johnsen et al. (2016)
Incubation	Co-incubation of drugs with secretory cells or exosomes	Simple operation, strong repeatability, no change in drug activity, no damage to the structural integrity of the secretion	Low loading efficiency, exosomes need to be isolated and purified from co-incubates again	Wang et al. (2022)
Freeze-thaw cycle	Mix the drug with exosomes, freeze quickly, thaw at room temperature, and perform freeze-thaw cycle	Simple operation without damaging the integrity of the exocrine membrane	Limited loading efficiency, may lead to exosome aggregation	Sato et al. (2016)
Extrusion	Mixing of drug with exosomes added to a syringe-based lipid extruder	Simple operation, high drug loading capacity	Impairs the structural integrity of the exosomal membrane, and may cause loss of surface proteins responsible for target delivery	Wan et al. (2018)
Sonication	The drug is mixed with exosomes and then sonicated to form transient holes	High load efficiency and large drug load	Impairs the structural integrity of the exosomal membrane, and may cause loss of surface proteins responsible for target delivery	Kim et al. (2016)
Electroporation	Applying an electric field to an exosome in a conductive solution, thereby creating holes in the exosome membrane	Preferred for loading macromolecules with high efficiency of loading hydrophilic drugs, and no chemical reagents are introduced	Impairs the structural integrity of the exosomal membrane, high-voltage pulses cause exosome cleavage	Johnsen et al. (2016)
Conjugation	Attachment of drugs to exosome membranes by click chemistry	High efficiency, enhanced exosome targeting	Complex operation, can only load molecules containing specific functional groups	Lee et al. (2018)
Microfluidics	Exosomes were captured in real time and modified by microfluidic chips	High throughput, fully automated, small sample size required	High chip cost, and co-precipitation of impurities during exosome and microfluidic chip manufacturing	Jo et al. (2014)

## Role of exosomes in the pathogenesis

### Keratonosus

The cornea is the outermost anatomical structure of the eye, exposed to the external environment. Corneal-related diseases are mainly corneal trauma caused by chemical or physical damage, associated with inflammation, ulceration, corneal scar, and neovascularization (Whitcher et al., 2001). If not treated effectively in time, blindness may result. The pathogenesis of corneal fibrosis/scar formation is characterized by excessive production of extracellular matrix (ECM) and deposition in the stroma layer. ECM remodeling can lead to corneal fibrosis, accompanied by inflammation and neovascularization (Janin-Manificat et al., 2012; Wilson et al., 2012). Exosomes derived from corneal fibroblasts can transport matrix metalloproteinase 14 to vascular endothelial cells, thus mediating corneal angiogenesis (Han et al., 2015). The role of exosomes in the physiological and pathological reactions of corneal wound healing and scar formation has been extensively studied (Samaeekia et al., 2018; McKay et al., 2020; Yeung et al., 2022). *In vitro* experiments have demonstrated that mouse corneal epithelium-derived exosomes fuse with corneal stromal cells and induce myofibroblast transformation, leading to the formation of corneal scar (Han et al., 2017). These results suggest that exosomes are important mediators of corneal wound healing, play a role in the intercellular communication between epithelium and stroma, and may serve as therapeutic targets for corneal tissue regeneration.

### Glaucoma

High IOP has long been considered as the most important risk factor for the development and progression of glaucoma (Vidal-Sanz et al., 2012). Exosomes can participate in regulating the function of trabecular meshwork cells, including involvement in transporting the glaucoma-associated secretory protein myocilin (Stamer et al., 2011), interfering with the classical Wnt signaling pathway (Tabak et al., 2018), regulating ECM turnover and remodeling (Dismuke et al., 2016), regulating atrial aqueous dynamics, thereby affecting intraocular pressure, thus participating in the development of glaucoma. Further exploration of the exosome pathway will help to find new targets for glaucoma treatment targeting the aqueous humor drainage system. Exosome-mediated inflammation also plays an important role in the pathogenesis of glaucoma (Aires et al., 2020). When intraocular pressure increases, exosomes derived from retinal microglia interact with receptor microglia cells to induce neuroinflammation, inducing oxidative stress and retinal ganglion cells (RGCs) death, leading to retinal degeneration of glaucoma.

### Diabetes retinopathy

Diabetic retinopathy (DR) is a microvascular complication of diabetes mellitus. It is caused by long-term chronic hyperglycemia that leads to leakage and obstruction of the retinal microvascular, resulting in fundus lesions. DR has a complex pathogenesis and can result in irreversible vision loss. Exosome levels in blood were higher in diabetic rats and diabetic patients than in normal controls (Huang et al., 2018; Mazzeo et al., 2018), and the increased number of exosomes reflects the increased secretory activity of cells in the state of diabetes, which may help accelerate the progression of diabetic retinopathy (Ogata et al., 2005). In addition, exosomes transport inflammatory factors, activate endothelial cells, and participate in angiogenesis and diabetic microangiopathy under certain conditions.

In terms of vascular injury, endothelial cells are directly exposed to blood, and several studies have shown that endothelial cells can take up circulating exosomes (Zhang et al., 2018; Yao et al., 2019). Hang et al. demonstrated that the complement content of plasma exosomes increased in patients with diabetic retinopathy, and exosomes rich in IgG could promote the activation of the diabetes-induced classical complement pathway, and the up-regulation of pro-inflammatory factors and chemokines, thus leading to vascular damage and eventually causing an increase in retinal microvascular leakage. Conversely, IgG-deficient exosomes reduce retinal vascular leakage, suggesting that a complement cascade that inhibits exosomal activation may prevent the progression of diabetic retinopathy (Huang et al., 2018). Through proteomic analysis, Zhang et al. (2018) found a significant increase in Arginase 1 content in serum exosomes of diabetic mice. These exosomes rich in Arginase 1 were taken up by endothelial cells, inhibiting the production of nitric oxide, and damaging the function of vascular endothelial cells.

In terms of neovascularization, Tokarz et al. (2015) compared plasma EVs with plasma without EVs in diabetic patients, and found that cytokines and angiogenic factors were significantly increased in EVs in diabetic patients, suggesting that exosomes promote angiogenesis, by transporting pro-angiogenic factors to accelerate the progression of diabetic retinopathy. On the other hand, some studies have shown that plasma exosomes from diabetic patients also contain components that inhibit angiogenesis. Xiong et al. (2020) extracted exosomes from the blood of patients with type 2 diabetes and observed that miR-20b-5p was significantly up-regulated. The miRNA could inhibit the angiogenesis of human umbilical vein endothelial cells by regulating Wnt9b/β-catenin signaling.

These results suggest that the content or composition of exosomes changes with diabetes, and plays an important role in the retinal vascular disease of diabetes. Given that blood is a kind of tissue that can reach the whole body, the cellular origin of plasma exosomes is relatively complex, and the heterogeneity of exosomes may be one of the reasons for their different effects. Further studies are needed to determine how exosomes precisely regulate vascular injury and neovascularization under specific conditions.

### Age-related macular degeneration

The progressive degeneration and death of retinal pigment epithelial (RPE) cells are considered as the initial pathology of age-related macular degeneration (AMD), and oxidative stress is considered as an important factor leading to RPE injury and degeneration. Early AMD is characterized by the formation of drusen, which is closely related to the RPE cells. Zhang et al. (2019a) showed that after oxidative stress injury induced by blue light, the RPE can secrete exosomes rich in IL-1β, IL-18, and caspase-1, which in turn can activate NLRP3 inflammasome and further aggravate the oxidative stress response of RPE. Compared with normal RPE, RPE damaged by oxidative stress secreted increased exosomes and carried vascular endothelial growth factor (VEGF) receptors 1 and 2, which promoted endothelial cell neovascularization (Atienzar-Aroca et al., 2016; Tong et al., 2016). Complement factor H (CFH) dysfunction is associated with AMD. Wang et al. proposed that in aging retinal tissue, CFH dysfunction in the complement pathway can lead to RPE-derived exosomes containing C3 molecules becoming targets of invading leukocytes and being attacked by leukocytes, leading to instability of the exosome membrane and release of intracellular proteins, thus promoting the formation of drusen. In addition, they also found increased expression of exosome markers CD63, CD8, and LAMP2 in AMD patients with drusen, but not in age-matched healthy controls (Wang et al., 2009). The above findings indicate that RPE-derived exosomes are involved in the development of AMD, suggesting that exosomes interact with the complement pathway, and RPE-derived exosomes may be targets of the complement system.

## Exosomes can be employed as a diagnostic biomarker

### Glaucoma

Early symptoms of primary glaucoma are insidious, and nearly half of Chinese glaucoma patients have missed the best time for treatment when diagnosed (Liang et al., 2018). In addition to conventional diagnostic methods, exosomes can be used as risk predictors to supplement the existing glaucoma diagnostic strategies. Sacca et al. (2012) found through proteomic analysis that compared with healthy controls, the expression levels of several exosome proteins in aqueous humor of glaucoma patients were significantly increased. Among them, heat shock protein 90 played a key role in the release of exosomes (Lauwers et al., 2018). Dismuke et al. (2015) detected miRNAs involved in aqueous humor generation and drainage tissue communication, such as miR-204, miR-191, and miR-148a, suggesting that glaucoma can be diagnosed by identifying the content of specific miRNAs in aqueous exosomes.

### DR

Exosomes are also important for the diagnosis of DR. The change of exosome contents is earlier than the appearance of fundus lesions, and has high specificity. It is an ideal biological marker for early diagnosis of DR, prediction of disease progress, and prognosis. Sadeghzadeh et al. (2020) proposed that the plasma expression levels of miR-222 and miR-15a can be used to distinguish pre-diabetes from normal people. Compared with diabetes, the expression of miR-15a in pre-diabetes patients is significantly downregulated. Compared with healthy controls, the miRNA expression profile of plasma EVs in diabetes was different. Mazzeo et al. (2018) found that the expression of miR-150-5p, miR-21-3p, and miR-30b-5p in the diabetes group was increased, which can be used as a potential biomarker of diabetes retinopathy. Further, Fan et al. analyzed the mRNA of plasma exosomes in type 1 diabetes patients and healthy controls, and found four differentially expressed mRNA with statistical significance, indicating that plasma-derived exosomes mRNAs can be used as a new minimally invasive diagnostic tool for diabetes.

## Role of exosomes in treatment

### Keratonosus

**FIGURE 4:**
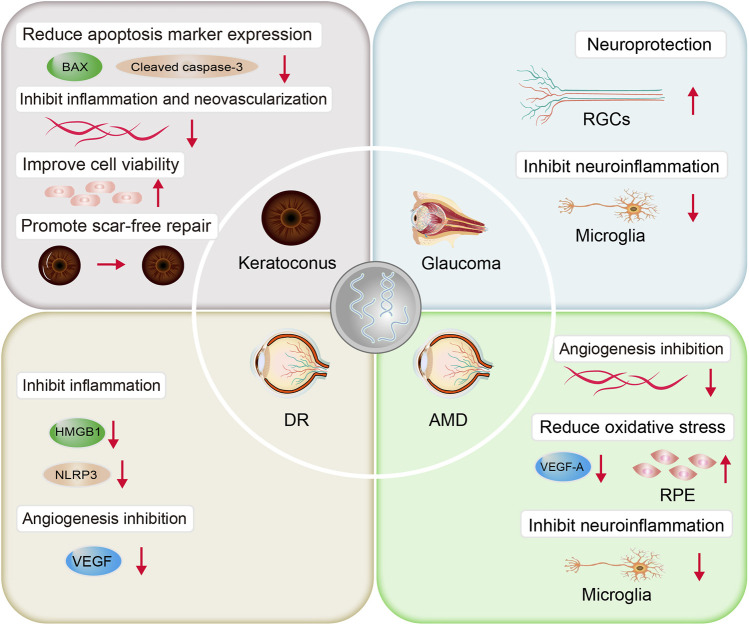
Mechanism of therapeutic action of exosomes in ocular diseases.

Several studies have demonstrated the therapeutic effect of stem cell-derived exosomes on the corneal injury (Hertsenberg et al., 2017; Shojaati et al., 2019; Ma et al., 2022). In the corneal injury mouse model, combined with an autophagy activator, human umbilical cord MSC-derived exosomes (MSC-exos) activated the autophagy AMPK-mTOR-ULK1 signaling pathway, reduced the levels of the apoptotic markers, Bax and cleaved Caspase-3, and decreased the expression of the inflammatory response products, TNF-α, IL-1β, IL-6, and CXCL-2, thereby reducing corneal epithelial defects and stromal opacity (Figure 4). *In vitro* experiments showed that the combination treatment significantly increased the viability of human corneal epithelial cells and promoted cell proliferation (Ma et al., 2022). Hertsenberg et al. (2017) demonstrated that exosomes from human corneal stromal stem cells through a TSG-6-dependent pathway, down-regulate the expression of the fibrosis markers myostatin C, Acta2, and Col3a1, inhibit neutrophil infiltration, inhibit corneal inflammation, stimulate regeneration of transparent stroma, and reduce corneal scar formation and fibrosis. Further experiments demonstrated that the above effects were produced by miRNA-dependent mechanisms. Gene knockdown using siRNA reduced miRNA in exosomes by 85%, and the effect of exosomes to prevent corneal scar formation was subsequently lost (Shojaati et al., 2019). Based on microfluidic technology, Yu et al. (2022) introduced human corneal-derived cells, simulated the environment of corneal injury with microfluidics, designed and constructed an *in vitro* bionic corneal chip, and found that stem cell exosomes could significantly promote the recovery of corneal epithelium, reduce the expression of matrix metallopeptidase-2 protein, inhibit corneal inflammation and neovascularization, and facilitate the process of injury repair. The above studies demonstrated that stem cell exosomes promote scar-free repair of corneal injury and help maintain corneal transparency. Meanwhile, different engineering strategies can be used to load exosomes with therapeutic components [such as miRNA, lncRNA (Sun Y. et al., 2018)] and construct exosome scaffolds to further enhance their therapeutic effects.

### Glaucoma

Stem cell-derived exosomes can be an effective neuroprotective and regenerative therapy (Doeppner et al., 2015). Using bone marrow MSC-exos, Mead et al. demonstrated that exosomes preserve RGCs function and promote RGCs survival and axonal regeneration through miRNA-dependent mechanisms. Exosomes deliver miRNA to RGCs for expression, leading to the translation of new proteins. After the key effector molecule Argonaute-2 of miRNA is knocked out, the therapeutic effect of exosomes is weakened (Mead and Tomarev, 2017); Liu et al. (2022) demonstrated that exosomes inhibit microglial activation and its associated neuroinflammation, and up-regulate synapse-associated protein and brain-derived neurotrophic factor protein expression. In summary, bone marrow MSC-exos can act as neuroprotective agents in glaucoma, promoting RGCs survival, inhibiting neuroinflammatory damage, and delivering neurotrophic proteins. In addition, umbilical cord MSC-exos (Pan et al., 2019) and human embryonic stem cell-derived exosomes (Seyedrazizadeh et al., 2020) also showed neuroprotective effects.

### DR

In the treatment of diabetic retinopathy, MSC-exos inhibit the inflammatory response and the formation of new blood vessels. Pakravan et al. (2017) demonstrated that MSC-exos significantly inhibited VEGF expression and secretion in a dose-dependent manner. Moreover, MSC-exos inhibit hyperglycemia-induced inflammatory responses, and MSC-exos overexpressing miR-126 significantly reduced HMGB1 expression and NLRP3 inflammasome activity in hyperglycemia-treated retinal endothelial cells (Zhang et al., 2019b), suggesting that miR-126 in MSC-exos reduces hyperglycemic-induced retinal inflammation by down-regulating the HMGB1 signaling pathway.

### AMD

Choroidal neovascularization (CNV) is an important cause of blindness in AMD patients and causes mechanical compression of RPE (Farjood et al., 2020). Hajrasouliha et al. (2013) found that subconjunctiva injection of retinal astrocyte-derived exosomes (RAC-exos) targeted both macrophages and vascular endothelial cells, inhibited their infiltration and migration, and had anti-angiogenic effects, whereas exosomes from RPE or non-retinal tissue cells did not show such anti-angiogenic effects. Although intravitreal injection of anti VEGF is an effective treatment, subconjunctival injection is less invasive than intravitreal injection (Kim and Woo, 2021). Meanwhile, subconjunctival exosomes injection allows for rapid drug delivery to the choroid and retina, which may be used for anti angiogenesis treatment of wet AMD in the future. The above experiments suggest that subconjunctival exosome injection may be a safer and more effective way of drug delivery. Zhu et al. (2020) further showed that normal RAC-exos were able to significantly inhibit angiogenesis, whereas under oxidative stress conditions, RAC autophagy levels were elevated and exosomes derived from them had the opposite effect, manifesting as promotion of human umbilical vein endothelial cell metastasis and angiogenesis.

Stem cell-derived exosomes have also been shown to have therapeutic effects. He et al. (2018) demonstrated that cord blood MSC-exos downregulated VEGF-A, alleviating blue light and laser-induced retinal pigment epithelial damage. On the other hand, mouse neural progenitor cell-derived exosomes can inhibit microglia activation. The miRNAs carried by exosomes inhibit inflammatory signaling pathways in microglia by reducing the expression of TNF-α, IL-1β, and COX-2, thus reducing photoreceptor apoptosis *in vivo* or *in vitro*, delaying photoreceptor degeneration and preserving visual function (Bian et al., 2020).

It should be emphasized that exosomes are not only involved in the above-mentioned ocular diseases. The biological role of exosomes in other ocular diseases is summarized in Table 2.

**TABLE 2:** The role of exosomes in other ocular diseases.

Ocular disease	Origin of exosomes	Role in ocular diseases	Example	References
Myopia	Aqueous humor	Diagnosis	15 specific miRNAs and 4 missing miRNAs were detected, and 6 myopic-related genes were identified	Chen et al. (2019)
Sjogren’s syndrome	T cells	Pathogenesis	The miR-142-3p in exosomes impairs epithelial cell function, causing glandular inflammation and dryness	Cortes-Troncoso et al. (2020)
Sjogren’s syndrome	Minor Salivary Gland	Diagnosis	Circ-IQGAP2 and Circ-ZC3H6 were significantly up-regulated and could be used as diagnostic biomarkers	Li et al. (2020)
Sjogren’s syndrome	mesenchymal stem cells	Treatment	Significantly reduces dry eye symptoms and lacrimal gland inflammation in rabbits	Li N. et al. (2019)
Autoimmune uveitis	mesenchymal stem cells	Treatment	Reduces infiltration of T-cell subsets and inflammatory cells	Bai et al. (2017)
Autoimmune uveitis	Regulatory B cells	Treatment	Save the function of photoreceptors	Kang et al. (2020)
Thyroid associated ophthalmopathy	Peripheral blood mononuclear cells	Pathogenesis	Increased mRNA expression of IL-1β, TNF-α	Hiratsuka et al. (2016)
Neovascular age-related macular degeneration	Aqueous humor	Diagnosis	Exosomes and their proteins are expected to become potential biomarkers	Kang et al. (2014)
Age-related macular degeneration	Serum	Diagnosis	The expression of miR-486-5p, miR-626 increased, and miR-885-5p decreased	Elbay et al. (2019)
Proliferative diabetic retinopathy	Aqueous humor and vitreous fluid	Diagnosis	Increased concentration of peroxisomeproliferator activated receiver gamma	Katome et al. (2015)
Retinopathy of Prematurity	Microglia	Treatment	Reduce photoreceptor damage and inhibit neovascularization	Xu et al. (2019)
Uveal melanoma	Vitreum, serum	Diagnosis	miR-146a expression is significantly upregulated and can be used as a diagnostic marker	Ragusa et al. (2015)

### Exosomes for precision medicine: Opportunities and challenges

MSCs have promising clinical translational applications (Luo Z. et al., 2021; Luo et al., 2022b). MSCs exosomes are safe, stable, easy to store, non-immunogenic, small in size, easily penetrate the human biological barrier, can be modified and engineered to act efficiently on target cells, and may serve as a non-cellular therapeutic biologic drug, and in the long run, a new alternative to MSC therapy (EL Andaloussi et al., 2013; Guillamat-Prats, 2021). Several studies mentioned above demonstrated that exosomes carry and transfer their contents such as miRNAs, proteins for intercellular communication and generate the corresponding biological functions. As the content of protein in the exosomes reaches the therapeutic dose and has sufficient functional activity, the ability of protein to initiate biological activity is widely recognized (Toh et al., 2018). Quantitative analysis of the stoichiometry of exosomal miRNAs showed that there is less than one copy per exosome (Chevillet et al., 2014). This implies that natural endogenous exosomes in a physiological setting lack the potential to act as therapeutic potential for miRNA transfer mediators. However, studies of exosome function often use large excesses of exosomes. Stevanato et al. (2016) demonstrated that exosomes can be mesoenriched with certain selected miRNAs up to 10 copies per exosome, and that exosomal agents can transfer certain miRNAs at a functional level to mediate biological effects.

As a natural nanoscale vesicle secreted by cells, exosomes are considered to be drug delivery vehicles with minimal immune response. Based on the exosome engineering strategy, hydrophobic substances can be inserted into the bilayer lipid membrane of the exosome, ensuring the therapeutic activity of the drug without chemical modification. In addition, natural exosomes derived from M2 type macrophages have anti-inflammatory properties. Li et al. (2022) engineered the exosomes derived from M2 type macrophages, transfected plasmids, and loaded with anti-inflammatory drugs. Through the synergistic effect of multiple mechanisms, macrophages were induced to transform from pro-inflammatory M1 type to anti-inflammatory M2 type. Engineered exosomes showed good inflammatory inhibition and targeted enrichment of inflammatory sites in inflammatory diseases, which could be used as a reference for inflammatory diseases in ophthalmology. In terms of targeted therapy, engineered exosomes, as targeting agents, have been proven to directly and specifically target oncogenes, significantly inhibit cancer cell proliferation, minimize cytotoxicity, and prolong the survival of pancreatic cancer mice (Kamerkar et al., 2017). Because the eye is a very small organ and its special anatomy, intravenous drug delivery has low drug utilization, and the application of engineered exosomes for targeted research in tumors has important implications for targeted therapy in ophthalmology.

At the same time, the nanoscale size allows exosomes to penetrate the human biological barrier, which has good retinal penetration. They can be injected through the vitreous cavity to reach the inner and outer layers of the retina (Pollalis et al., 2022), and can also be injected through the peribulbar quickly cross the wall of the eye to reach the eye (Hajrasouliha et al., 2013), with a better therapeutic effect than systemic intravenous injection (Safwat et al., 2018). Biocompatible scaffolds such as hydrogel dressings can greatly increase the retention rate and stability of exosomes *in vivo*. Tang et al. (2022) developed an exocrine sustained-release system based on thermosensitive chitosan hydrogel, which proved that it could effectively promote the repair and regeneration of damaged corneal epithelium and stroma, accelerate the corneal wound healing process, and reduce the formation of corneal scar. The above studies suggest that exosomes have great potential as intraocular drug delivery carriers, and the construction of exosome carriers can realize pulse therapy with a high concentration of drugs in the eye, reducing adverse effects and improving therapeutic efficacy.

While exosomes are significantly heterogeneous (Chiang and Chen, 2019), and quality control is a pressing challenge. But there is a lack of unified research standards, and there is an urgent need for more standard isolation and purification, analytical methods, and engineering strategies to obtain highly pure and consistent exosomes, to deeply explore valuable information about their biological functions. In addition, achieving mass production of therapeutic exosomes for clinical use is a challenge (van der Meel et al., 2014). The use of exosomes in the diagnosis of eye diseases is rarely studied, and false negative results may be caused by low concentrations of exosomes in body fluids (Sun Z. et al., 2018). However, microfluidic technology, which can rapidly and sensitively analyze exosomes, provides a miniaturized, integrated, and automated diagnostic method. With the development of modern technology and high-throughput sequencing facilities, exosomes as specific markers of eye diseases will be more widely studied and applied.

## Conclusion

Exosomes play an important role in various biological processes, affecting various physiological and pathological processes. It has been proved that exosomes are involved in the pathogenesis, diagnosis, and treatment of ophthalmopathy from the molecular mechanism. In the future, the construction of an exosome delivery and treatment platform based on engineered exosomes is conducive to the realization of precision medicine for ocular diseases.
